# Linear Birefringent Films of Cellulose Nanocrystals Produced by Dip-Coating

**DOI:** 10.3390/nano9010045

**Published:** 2018-12-31

**Authors:** Arturo Mendoza-Galván, Tania Tejeda-Galán, Amos B. Domínguez-Gómez, Reina Araceli Mauricio-Sánchez, Kenneth Järrendahl, Hans Arwin

**Affiliations:** 1 Cinvestav-Querétaro, Libramiento Norponiente 2000, 76230 Querétaro, Mexico; tania.tejeda@cinvestav.mx (T.T.-G.); bdominguez@cinvestav.mx (A.B.D.-G.); amauricio@cinvestav.mx (R.A.M.-S.); 2 Materials Optics, Department of Physics, Chemistry and Biology, Linköping University, SE-58183, Linköping, Sweden; kenneth.jarrendahl@liu.se (K.J.); hans.arwin@liu.se (H.A.)

**Keywords:** nanostructured films, birefringence, nanocrystalline cellulose, Mueller matrix

## Abstract

Transparent films of cellulose nanocrystals (CNC) are prepared by dip-coating on glass substrates from aqueous suspensions of hydrolyzed filter paper. Dragging forces acting during films’ deposition promote a preferential alignment of the rod-shaped CNC. Films that are 2.8 and 6.0 µm in thickness show retardance effects, as evidenced by placing them between a linearly polarized light source and a linear polarizer sheet in the extinction configuration. Transmission Mueller matrix spectroscopic ellipsometry measurements at normal incidence as a function of sample rotation were used to characterize polarization properties. A differential decomposition of the Mueller matrix reveals linear birefringence as the unique polarization parameter. These results show a promising way for obtaining CNC birefringent films by a simple and controllable method.

## 1. Introduction

Cellulose is the most abundant renewable biopolymer on earth. Its polymeric chain of d-anhydroglucopyranose units, through a hierarchical arrangement, leads to a fibrous macroscopic structure with a semicrystalline character [1]. The extraction of cellulose nanocrystals (CNC) from cellulose fibrils through controlled, sulfuric acid-catalyzed degradation was reported more than half a century ago [2]. Since then, the needle-shaped CNC and their functionalization capabilities have found applications in diverse fields [3,4,5]. Besides removal of the amorphous regions, the sulfuric acid hydrolysis of cellulose fibrils functionalizes the CNC surface, resulting in negatively charged sulphate groups. The electrostatic interaction between charged CNC leads to stable aqueous suspensions. Decades ago, it was discovered that above a critical concentration, the CNC self-assemble in a chiral nematic liquid crystalline phase [6]. The slow drying of these aqueous suspensions produces films with helicoidal ordering that reflect left-handed polarized light [7,8]. Circular dichroism and circular birefringence are the characteristic polarization properties of chiral CNC films [8]. The spectral location and strength of this so-called circular Bragg reflection depend on the value and distribution of the helicoidal pitch, as well as on the birefringence of CNC.

The linear birefringence of cellulose-based fibers is known only at some wavelengths [9,10]. On the other hand, the birefringence of cellulose derivatives in film form has been studied extensively [11]. In recent times, interest in investigating the birefringence of CNC has increased [12,13,14,15,16,17,18,19,20]. Particularly, methods for the fabrication of birefringent CNC films have been focused on producing an effective alignment of CNC along a preferential direction. Spin-coating [15] and shear-ordering [16,17,18,19,20] methods have been reported. The development of new methods to fabricate birefringent CNC films will contribute to a better understanding of their fundamental properties and open new opportunities for novel applications. Specifically, thinking in optical biomimetics, they could be used as retarders sandwiched between chiral layers also made from cellulose nanocrystals. With an adequate thickness, the retardation could be tuned to manipulate left- and right-handed circular polarization in a specific spectral range.

In this work, we show that a simple and non-expensive dip-coating technique is suitable to prepare transparent and birefringent CNC films from aqueous suspensions. For a complete characterization of the polarization properties of the films, Mueller-matrix transmission spectroscopic ellipsometry is used. A unique capability of this approach is that the depolarization introduced by the sample, that is, how much the sample affects the degree of polarization of incident light, is also provided. Furthermore, as CNC in aqueous suspension tend to self-assemble in a chiral nematic liquid crystal phase, circular dichroism and circular birefringence could be expected. Therefore, a differential (logarithmic) decomposition of the Mueller matrix data was performed to determine the basic polarization properties of the dip-coated CNC films, including both linear and circular birefringence, as well as dichroism.

## 2. Materials and Methods

Aqueous suspensions of CNC were obtained following procedures as reported in the literature, but with slight variations [17,21,22]. Filter paper (Whatman 40) was grinded in a coffee mill by four cycles, 35 s each. The milled paper was hydrolyzed at 60 °C for 50 min under vigorous stirring using 64 wt% sulfuric acid at a ratio of 8.75 mL per 1 g of filter paper. To stop the hydrolysis, the CNC suspension was diluted with cold water (10 times the volume of the acid solution) and allowed to settle for two weeks. The clear top layer was decanted, and the remaining cloudy layer was subject to three cycles of centrifugation (9000 rpm for 10 min) and washing with water to remove water-soluble cellulose materials. The thick white suspension was dialyzed against water for three days. The initial concentration of the CNC suspension was 6.5 wt%, and it was diluted with water to attain a concentration of 5.7 wt%, suitable for dip-coating. Ultrasonic dispersion was not applied. Glass slides 25 × 75 mm (Corning 2947) washed with detergent were used as substrates. CNC films were then produced by dip-coating at withdrawal speeds of 10 and 20 cm/min by using a home-made apparatus. Subsequently, the samples were vertically placed and allowed to dry at room temperature for about 3–4 h.

Transmission Mueller-matrix measurements were performed with a dual rotating compensator ellipsometer (RC2, J. A. Woollam Co., Inc., Lincoln, NE, USA) at normal incidence in the wavelength (*λ*) range 210–1690 nm. Data are here presented versus photon energy given by *E* = *hc*/*λ*, where *h* is Planck’s constant and *c* the vacuum speed of light. A motorized sample rotator was used to measure at rotation angles between 0° and 360° in steps of 5°. Transmittance irradiance measurements at normal incidence in the spectral range of 250–840 nm were performed with a FilmTek 3000 system (SCI, Inc., Carlsbad, CA, USA). Atomic force microscopy (AFM) images in tapping mode were acquired with an Innova system (Bruker, Madison, WI, USA). Complementary characterization included X-ray diffraction data (Rigaku/Dmax2100, Austin, TX, USA), attenuated total reflection (ATR) infrared spectroscopy measurements (Spectrum GX system/Perkin Elmer Inc., Waltham, MA, USA), and cross-sectional scanning electron microscopy (SEM) (Phillips XL 30 system, North Billerica, MA, USA). To avoid charging during acquisition of SEM images, a thin layer of gold was deposited on the sample using Ar as carrier gas, 20 µA, 5 × 10^−2^ torr for 30 s (Denton Vacuum desk V, Moorestown, NJ, USA).

## 3. Results and Discussion

### 3.1. Formation of Nanostructured Films and Optical Performance

At the concentration of the CNC which was used (5.7 wt%), the coexistence of isotropic and anisotropic phases in the suspension was expected, as reported for hydrolyzed filter paper at similar conditions [21,22]. Therefore, we hypothesized that by using the dip-coating technique, the drag of draining forces acting during the removal of the substrate could align the nanocrystals in the isotropic phase and partially unwind the cholesteric order. As the substrate leaves the suspension, the draining of water produces an increase of CNC concentration in the entrained suspension, which limits the mobility of CNC. At the withdrawal speeds used, the coating process took about 15–30 s. During the drying stage, the draining of remaining water in the films promoted further alignment of CNC for about 30 min. The film then reached a gel-like state, where the CNC were frozen in a partial nematic ordering. At this stage, the film loses water by evaporation for about 2–3 h. Notice the difference in time scale between the dip-coating process and the evaporation-induced self-assembly of chiral films, as the latter takes a few days.

**Figure 1:**
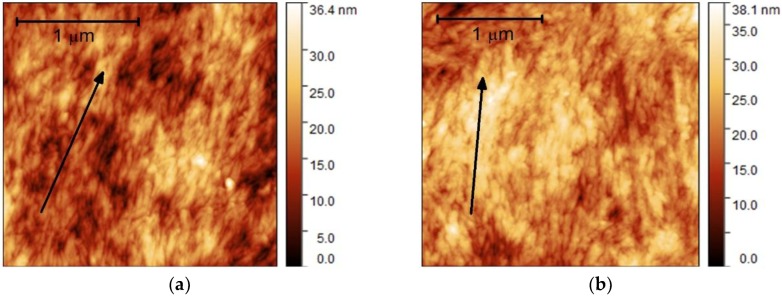
Atomic force microscopy (AFM) images of dip-coated cellulose nanocrystal (CNC) films on glass substrates produced at withdrawal speeds of (**a**) 10 and (**b**) 20 cm/min. The arrows illustrate the preferential ordering of CNC.

**Figure A1:**
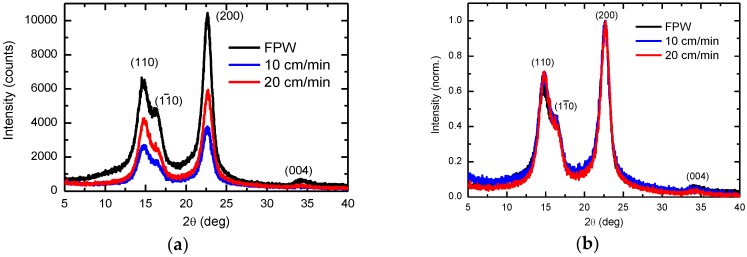
X-ray diffraction patterns of filter paper (FPW) and dip-coated CNC films at withdrawal speeds of 10 and 20 cm/min: (**a**) raw data (**b**) normalized to the (200) peak.

**Figure A2:**
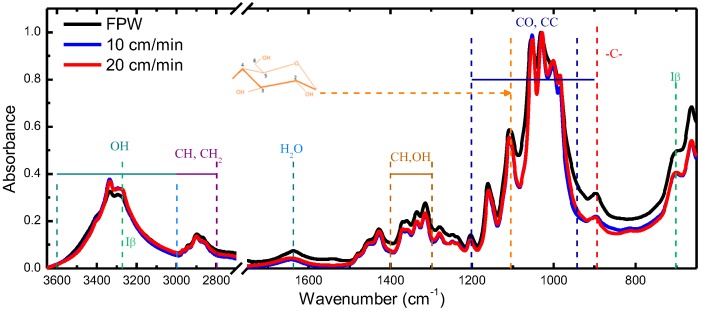
ATR infrared absorption spectra of filter paper (FPW) and dip-coated CNC films at withdrawal speeds of 10 and 20 cm/min

**Figure A3:**
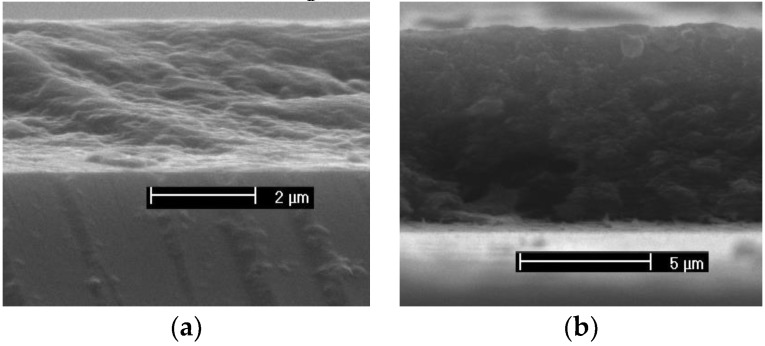
Cross-section SEM images of dip-coated CNC films at (**a**) 10 and (**b**) 20 cm/min.

Figure 1 shows AFM images of the surfaces of the dip-coated CNC films. A preferential alignment of the CNC is observed, and thus, our hypothesis is confirmed. X-ray diffraction data (see Figure A1 in Appendix A) corroborated that the films retain the monoclinic crystalline structure of cellulose Iβ. ATR infrared spectroscopy measurements (see Figure A2 in Appendix A) evidenced that the molecular integrity of the cellulose was retained as well. The thicknesses determined from cross-sectional SEM images (see Figure A3 in Appendix A) were 2.8 ± 0.1 and 6.0 ± 0.5 µm for 10 and 20 cm/min withdrawal speeds, respectively.

**Figure 2:**
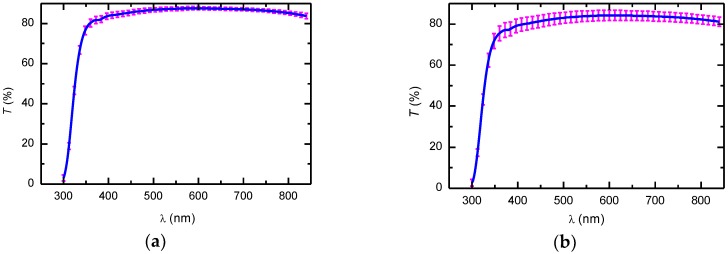
Average transmittance of unpolarized light of dip-coated CNC films on glass substrates at withdrawal speeds of (**a**) 10 and (**b**) 20 cm/min. Error bars are the standard deviation of three spectra.

The transmittance (*T*) spectra at normal incidence of unpolarized light from the cellulose side of the dip-coated CNC films are shown in Figure 2. The spectra correspond to the average of three measurements performed at different regions on the films. The standard deviation of the three measurements were plotted as error bars. As can be seen, the films show good transparency with *T* values above 80% in the visible spectral range. Interference oscillations are missing due to the low optical contrast at the cellulose–glass interface because the refractive indices of cellulose ~1.54 [10] and the glass substrate ~1.52 are similar. The decrease in *T* at wavelengths shorter than 350 nm is due to absorption of the glass substrate.

**Figure 3:**
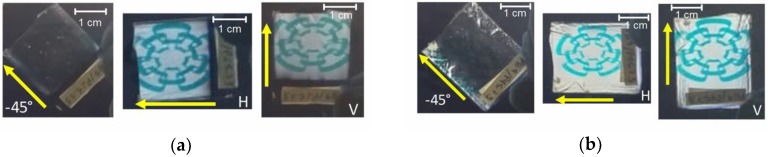
Pictures of dip-coated CNC films on glass substrates at withdrawal speeds of (**a**) 10 and (**b**) 20 cm/min placed between an LCD monitor and a polarizer in the extinction configuration without a sample. The arrows indicate the withdrawal direction. Sample orientation at +45° looks like −45°.

Qualitative and simple evidence of the birefringence of a sample is to place it between crossed polarizers. This can be done by using a liquid crystal display (LCD) as a source of linearly polarized light and a linear polarizer sheet. Figure 3 shows pictures of the assembly used. The plane of polarization of light coming from the LCD screen was at about +45° from the horizontal direction, and the polarizer sheet was set in the extinction configuration (see dark regions around the samples). As can be seen, when the polarization plane of the light coming from the LCD is perpendicular to the withdrawal direction (indicated with the arrows), the sample looks dark (sample orientation −45°). This means that the linear polarization is not affected by the sample, and the outcoming light is cancelled out by the polarizer sheet. On the other hand, by rotating the samples to the horizontal (H) and vertical (V) orientations, the image on the LCD screen can clearly be seen. In these configurations, which are the so-called maximum transmittance, the polarization state of the linearly polarized incident light is altered and, in general, exits the sample with elliptical polarization. Thus, the in-plane anisotropy in the films is evident, and its origin resides in the preferential ordering of CNC and in the intrinsic anisotropy of cellulose, as discussed in Section 3.4. Another salient feature is the homogeneity over large areas of the samples. However, some inhomogeneities can be seen at the edges of the substrates due to accumulated material by border effects. The draining also accumulates material at the bottom part of the substrates.

### 3.2. Mueller Matrix Data Analysis

The 4 × 4 Mueller matrix (**M**) with elements *m_ij_* (*i*, *j* = 1…4) provides a full description of the polarizing and depolarizing properties of a sample [23]. It relates the Stokes vectors of the incident (**S***_i_*) and transmitted (**S***_t_*) light beams by:

(1) $$S_{t}=MS_{i}.$$

In Equation (1), the Stokes vectors of the incident and transmitted light beams (assuming propagation along the *z*-axis) are expressed in terms of a set of six irradiances:

(2) $$S=\left[\begin{array}{c} I_{x}+I_{y}\\ I_{x}-I_{y}\\ I_{+45{}^{\circ}}-I_{-45{}^{\circ}}\\ I_{R}-I_{L} \end{array}\right],$$

where *I_x_*, *I_y_*, *I*_+45°_, and *I*_−45°_ correspond to linear polarization along the coordinate axes *x* and *y*, and at +45° and at −45° from the *x*-axis, whereas *I_R_* and *I_L_* correspond to right- and left-handed circularly polarized light, respectively. In this work we used Mueller matrices, which were normalized to total transmittance for unpolarized light, i.e., the first element in the first row of **M**. Thus, we have *m*_11_ = 1, and other elements have values in the range [−1,1]. For the measurements, the samples were placed with the withdrawal direction nearly parallel to the *y*-axis.

**Figure 4:**
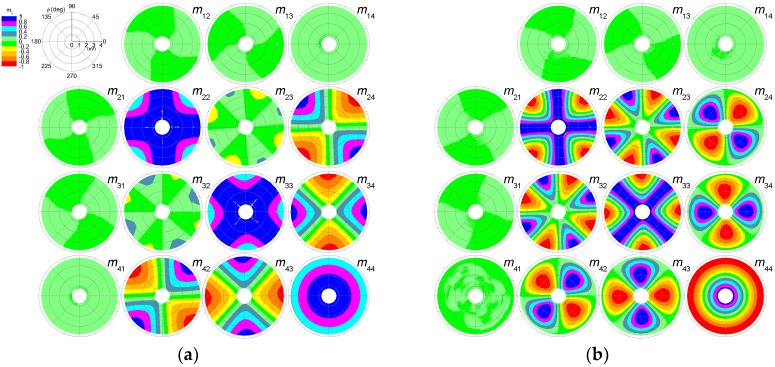
Polar contour maps of normal-incidence Mueller matrix transmission measurements for dip-coated films produced at withdrawal speeds of (**a**) 10 cm/min and (**b**) 20 cm/min.

Figure 4 shows polar contour maps of the normal-incidence Mueller matrix transmission data of dip-coated CNC films. The radial and angular coordinates correspond to the photon energy (in eV) and sample rotation angle (*ϕ*), respectively. Data above 3.75 eV (below λ = 330 nm) were omitted because the glass substrate strongly absorbs the ultraviolet range (*T* lower than 50% in Figure 2). First, we observed that the elements of the first row and first column were close to zero, whereas the other elements show a richer structure. Second, we found that **M** was not block diagonal, which it would be for an isotropic sample or for uniaxially samples with the optic axis aligned with the *z*-direction. In Figure 4, the non-zero off-diagonal elements *m*_42_ and *m*_24_ thus provide evidence of in-plane anisotropy. Other characteristics of **M** are related to the *ϕ*-dependence: (i) *m*_44_ is invariant; (ii) *m*_24_, *m*_42_, *m*_34_, and *m*_43_ are periodic with period 180°; (iii) a 90° periodicity is observed in *m*_22_, *m*_23_, *m*_32_, and *m*_33_; (iv) rotational shift relationships noted are *m*_22_(*ϕ*) = *m*_33_(*ϕ* − ±45°), *m*_34_(*ϕ*)=*m*_24_(*ϕ* − 45°), and *m*_43_(*ϕ*) = *m*_42_(*ϕ* + 45°). Other symmetries are also observed: *m*_23_ ≅ *m*_32_, *m*_24_ = −*m*_42_, and *m*_34_ = −*m*_43_. Furthermore, we noticed that increases in film thickness resulted in larger variations of the *m_ij_* values. As an example, the diagonal elements vary between 0 and 1 for the 2.8 µm-thick film, whereas the variation is between −1 and 1 in the case of the 6 µm-thick film. Indeed, the contour maps in Figure 4a look like a zoom of the central part of the corresponding ones in Figure 4b.

**Figure 5:**
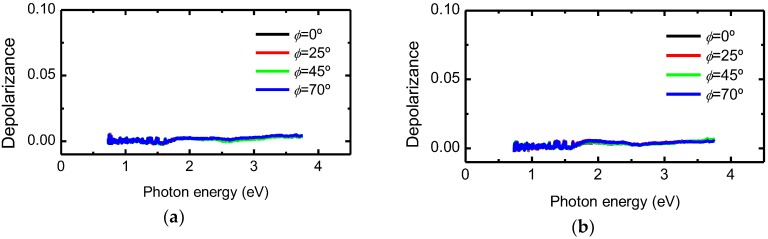
Depolarizance of normal-incidence Mueller matrices for dip-coated films produced at withdrawal speeds of (**a**) 10 cm/min and (**b**) 20 cm/min at selected rotation angles.

**Figure A4:**
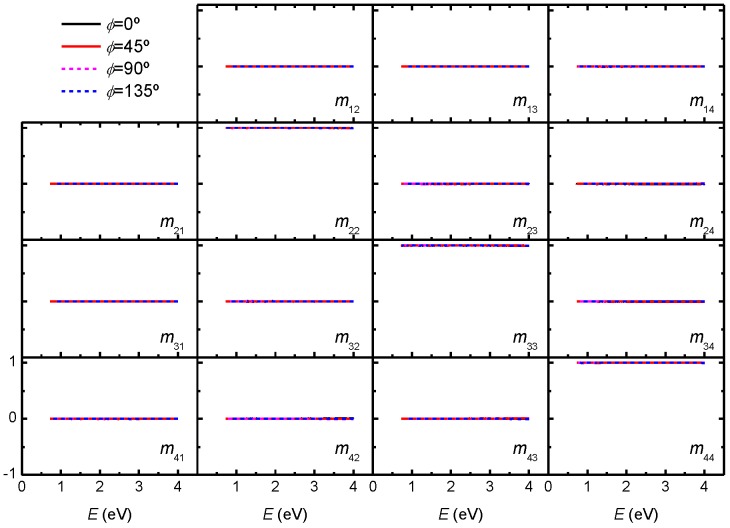
Mueller matrix of glass substrate measured at four rotation angles.

All the observations listed in the previous paragraph on the structure of **M** in Figure 4 come from the CNC films, because the Mueller matrix of the glass substrate measured at different rotation angles corresponds to the 4 × 4 unity matrix (see Figure A4 in Appendix A). Furthermore, **M** in Figure 4 qualitatively agrees with the Mueller matrix **M**_R_ of an ideal linear retarder plate in which the fast axis is horizontal, and is given by [23]:

(3) $$M_{R}=\left[\begin{array}{cccc} 1 & 0 & 0 & 0\\ 0 & \cos^{2}2\phi +\cos\delta \sin^{2}2\phi & \sin2\phi \cos2\phi \left(1-\cos\delta \right) & -\sin\delta \sin2\phi \\ 0 & \sin2\phi \cos2\phi \left(1-\cos\delta \right) & \sin^{2}2\phi +\cos\delta \cos^{2}2\phi & \sin\delta \cos2\phi \\ 0 & \sin\delta \sin2\phi & -\sin\delta \cos2\phi & \cos\delta \end{array}\right]\;,$$

where $\delta =2\pi \left(n_{y}-n_{x}\right)d/\lambda$ is the retardance, *d* the sample thickness, and *n_x_* and *n_y_* are the refractive indices for polarization along the *x*- and *y*-axes, respectively. However, as the matrices in Figure 4 were experimentally determined, it is necessary to investigate how much they deviate from those of ideal polarizing systems. This can be done by determining the depolarizance (*D*) of the system, which is given by [24]:

(4) D=1−[13(tr(MTM)m112−1)]12 ,

where *tr* and T stand for trace and transpose, respectively. It holds that *D* = 0 corresponds to the Mueller matrix of an ideal non-depolarizing system, and *D* = 1 to an ideal depolarizer. In Figure 5, *D* is shown for selected values of *ϕ*. As can be seen, the experimental Mueller matrices of the CNC films correspond very closely to those of ideal systems. This means that for totally polarized incident light, the transmitted beam emerges completely polarized.

### 3.3. Differential Decomposition of Mueller Matrices

To quantitatively determine the polarization properties of the dip-coated CNC films, we used differential (logarithmic) decomposition of the measured Mueller matrices. This decomposition establishes that **M** and its spatial variation along the direction of wave propagation *z* are related as dM/dz=mM, where **m** is the differential matrix [25,26,27,28]. For homogeneous media, **m** is independent of *z* and direct integration gives **L** = ln**M,** where **L** = **m***d* with *d* the sample thickness. The matrix **L** is split into $L=L_{m}+L_{u}$, where **L**_m_ and **L**_u_ are G-antisymmetric and G-symmetric matrices, respectively, given by $L_{m}=\left(L-GL^{T}G\right)/2$ and $L_{u}=\left(L+GL^{T}G\right)/2$, where G=diag[1,−1,−1,−1]. Since the dip-coated CNC films are non-depolarizing, it holds **L**_u_ = **0**. **L**_m_ contains the six elementary polarization properties, which are given by [26]:

(5) $$L_{m}=\left[\begin{array}{cccc} 0 & LD & LD^{\prime } & CD\\ LD & 0 & CB & -LB^{\prime }\\ LD^{\prime } & -CB & 0 & LB\\ CD & LB^{\prime } & -LB & 0 \end{array}\right]\;,$$

where *LB* (*LD*) and *LB*′ (*LD*′) are the linear birefringence (dichroism) along the *x*-*y* and ±45° axes, respectively, whereas *CD* is the circular dichroism and *CB* is the circular birefringence.

**Figure 6:**
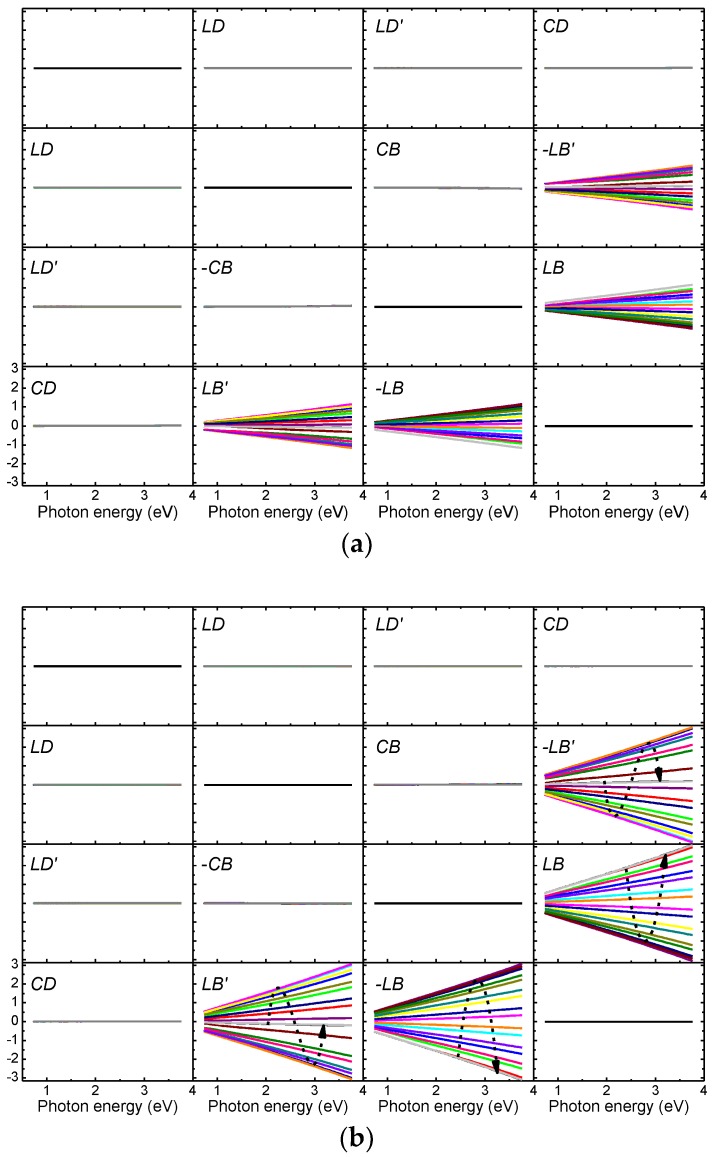
Differential decomposition of the Mueller matrices in Figure 4 for dip-coated films produced at withdrawal speeds of (**a**) 10 cm/min and (**b**) 20 cm/min. For clarity, data are presented only in the 0° ≤ *ϕ* ≤ 180° range in steps of 10°. The *ϕ*-variation is indicated with the dashed arrows in panels *LB* and *LB*′ of (**b**).

Figure 6 shows **L**_m_ corresponding to the experimental Mueller matrices in Figure 4. For the sake of clarity, data are only shown for 0° ≤ *ϕ* ≤ 180° in steps of 10°. For both samples, only *LB* and *LB*′ differ from zero, and both show a nearly linear dependence with photon energy. The small deviation from linearity implies a small dispersion in birefringence, as will be discussed later. Furthermore, *LB* increases with withdrawal speed (i.e., film thickness). The variation with rotation angle is illustrated in *LB* and *LB*′ panels in Figure 6b, and is analyzed in more detail in Section 3.4. The fact that *CD* and *CB* are zero indicates the absence of the chiral phase in the films.

### 3.4. Birefringence of Dip-Coated CNC Films

**Figure 7:**
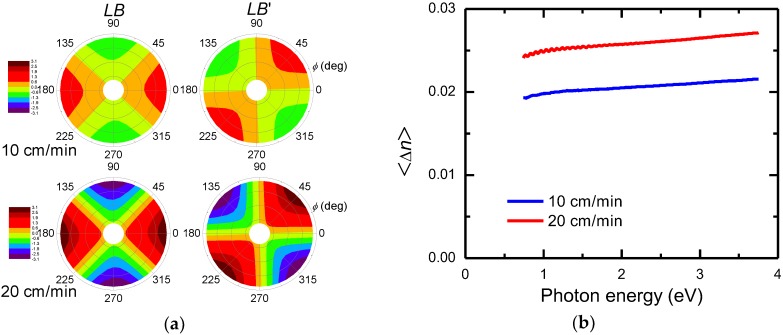
(**a**) Polar contour maps of linear birefringence of dip-coated CNC films at withdrawal speeds of 10 cm/min and 20 cm/min. The radial scale (photon energy) is the same as in Figure 4. (**b**) Effective birefringence <Δ*n*> = <*n_e_* − *n_o_*> of the dip-coated CNC films.

An insightful view of *LB*(*ϕ*) and *LB*′(*ϕ*) determined from the differential decomposition is provided when they are plotted in polar contour maps, as shown in Figure 7a where the radial coordinate corresponds to photon energy and the polar angle to sample azimuth *ϕ*. From Figure 7a, the obvious relationship *LB*′(*ϕ*) = *LB*(*ϕ* − 45°) is clear. Therefore, among the six basic polarization properties in Equation (5), only linear birefringence characterizes the dip-coated CNC films. The *ϕ*-dependence of *LB* (and *LB*′) can be expressed as [29], $LB\left(\phi \right)=\left|LB\right|\cos2(\phi -\phi_{0})$ and $LB\prime \left(\phi \right)=\left|LB\right|\sin2(\phi -\phi_{0})$, respectively, where *ϕ*_0_ is the azimuth offset between the *y*-axis and the optical axis of the sample. Values of *ϕ*_0_ = −2.2 and −1.9° were determined from the measurements at *ϕ* = 0° of the laboratory frame, as $\tan2\phi_{0}=-LB\prime \left(0\right)/LB\left(0\right)$. Since the sample birefringence $\left|LB\right|=2\pi \left|n_{y}-n_{x}\right|d/\lambda$, due to the preferential ordering of CNC the effective birefringence $\langle \Delta n\rangle =\langle n_{y}-n_{x}\rangle$ can be easily obtained and is shown in Figure 7b. The values determined for 〈Δn〉 are about half of those reported for cotton fibers [9,10].

According to the monoclinic crystalline structure of cellulose Iβ, its dielectric tensor corresponds to a biaxial crystal given as $\mathrm{diag}\left[\varepsilon_{1},\varepsilon_{2},\varepsilon_{3}\right]$ in the principal axes frame. Considering that the crystallographic *c*-axis corresponding to the polymer chain direction (crystallite length) is orthogonal to *a* and *b* axes, we have *ε*_3_ = *ε_c_* but *ε*_1_ and *ε*_2_ are not necessarily oriented along the *a* and *b* axes; besides, their orientation might be wavelength-dependent. Therefore, *n_y_* = *n_e_* would be mainly related to *ε_c_* and *n_x_* = *n_o_* to both *ε*_1_ and *ε*_2_, where *n_e_* and *n_o_* are the extraordinary and ordinary refractive indices, respectively. In the ideal case of perfect alignment and full packing, it is expected that *n_e_*^2^ = *ε_c_* and *n_o_*^2^ = (*ε*_1_ + *ε*_2_)/2, and we obtain the effective birefringence $\Delta n=n_{y}-n_{x}=n_{e}-n_{o}$. In the present case where the CNC comprising the films are not completely aligned, the structural birefringence will also come into play. In this case, the films could be envisaged as a two-component composite of aligned (anisotropic) and non-aligned (isotropic) phases. Thus, the effective birefringence would be a function of the volume fraction of the anisotropic phase. For the films studied in this work, 0.4–0.5 is a rough estimate of the anisotropic phase volume fraction, assuming a linear variation. Of course, the ordering of CNC crystals in the films depends on the various parameters intervening for their fabrication. In this regard, two main stages are identified: Preparation of the CNC aqueous suspension and films deposition. The physicochemical properties of the CNC suspension largely depend on parameters like hydrolysis temperature, hydrolysis time, acid ionic strength, among others. On the other hand, for films deposition, the withdrawal speed, CNC concentration, and drying conditions are of main importance. The large number of parameters open possibilities to fabricating CNC films with tailored birefringence, and is subject to ongoing investigation which will be reported elsewhere.

## 4. Conclusions

Birefringent cellulose nanocrystal films were obtained from aqueous suspensions of hydrolyzed filter paper at 60 °C for 50 min. The films prepared were transparent and non-depolarizing. The linear birefringence (*LB*) of the films was determined by a differential decomposition of Mueller matrices, and showed a nearly linear dependence with photon energy. The effective birefringence <Δ*n*> was due to the preferential ordering of CNC, and had average values in the visible range of 0.021 and 0.026 for two different film thicknesses. The absence of the chiral phase in the films was ascribed to the short drying time. The dip-coating method is thus shown to be a suitable, non-expensive, and promising way to fabricate birefringent films of cellulose nanocrystals.

## Appendix A

This Appendix contains additional figures supporting the results presented in the main text. Figure A1 shows X-ray diffraction data of the filter paper used as the source of cellulose and the dip-coated CNC films at withdrawal speeds of 10 and 20 cm/min. The Miller indices are those corresponding to the monoclinic structure of cellulose Iβ, with lattice parameters *a* = 7.8 Å, *b* = 7.9 Å, *c* = 10.1 Å, β = 95.15° and space group P2_1_ [30,31,32]. The differences in peaks intensities of the data in Figure A1a are due to film thickness as can be seen in Figure A1b when the data are normalized with respect to the most intense (200) peak. It can be noticed that the processing does not affected the crystalline quality of cellulose. The crystallite width evaluated with the Scherrer formula was 6 nm which is typical of cotton cellulose [32].

Figure A2 shows FTIR spectra of the filter paper used as the source of cellulose and dip-coated CNC films at withdrawal speeds of 10 and 20 cm/min. The spectra were normalized respect to the band at 1030 cm^−1^. As can be seen, the spectra of dip-coated CNC films and filter paper are similar indicating that processing does not affect the molecular integrity. The bands of functional groups in cellulose molecule are labeled as well as the characteristic bands identifying cellulose Iβ [32,33].

SEM images used to determine the film thicknesses of the dip-coated CNC films at withdrawal speeds of 10 and 20 cm/min are shown in Figure A3.

The normal incidence transmission Mueller matrices of a bare glass substrate measured at different rotation angles are shown in Figure A4. It can be noticed that they correspond to the 4 × 4 unity matrix.
